# Experimental Investigation of Thermal and Pressure Performance in Computer Cooling Systems Using Different Types of Nanofluids

**DOI:** 10.3390/nano9091231

**Published:** 2019-08-29

**Authors:** Altayyeb Alfaryjat, Lucian Miron, Horatiu Pop, Valentin Apostol, Mariana-Florentina Stefanescu, Alexandru Dobrovicescu

**Affiliations:** Faculty of Mechanical Engineering and Mechatronics, University Politehnica of Bucharest, Splaiul Independentei nr. 313, Sector 6, Bucharest 060042, Romania

**Keywords:** computer cooling system, nanofluids, microchannel heat sink

## Abstract

A modern computer generates a great amount of heat while working. In order to secure appropriate working conditions by extracting the heat, a specific mechanism should be used. This research paper presents the effect of nanofluids on the microchannel heat sink performance of computer cooling systems experimentally. CeO_2_, Al_2_O_3_ and ZrO_2_ nanoparticles suspended in 20% ethylene glycol and 80% distilled water are used as working fluids in the experiment. The concentration of the nanoparticles ranges from 0.5% to 2%, mass flow rate ranges from 0.028 kg/s to 0.084 kg/s, and the ambient temperature ranges from 25 °C to 40 °C. Regarding the thermal component, parameters such as thermophysical properties of the nanofluids and base fluids, central processing unit (CPU) temperature, heat transfer coefficient, pressure drop, and pumping power have been experimentally investigated. The results show that CeO_2_-EG/DW, at a concentration of 2% and a mass flow rate of 0.084 kg/s, has with 8% a lower temperature than the other nanofluids and with 29% a higher heat transfer coefficient compared with the base fluid. The Al_2_O_3_-EG/DW shows the lowest pressure drop and pumping power, while the CeO_2_-EG/DW and ZrO_2_-EG/DW show the highest. However, a slight increase of pumping power and pressure drop can be accepted, considering the high improvement that the nanofluid brings in computer cooling performance compared to the base fluid.

## 1. Introduction

In the last two decades, many electronic devices, such as high-power chips and LEDs, have appeared, and with them, high heat fluxes that need to be removed in order to maintain the electronic junctions at a normal operating temperature. A new way of removing a large amount of heat from small areas is through the use of a microchannel heat sink (MCHS), which was first presented by Tuckerman and Pease [1]. This idea was discovered based on the reverse relation between the characteristic length of the channel and the heat transfer coefficient. These devices are made of highly solid conductive materials, such as aluminium, copper, and silicone, provided with micro-channels for the passage of the cooling fluid. Moreover, a microchannel heat sink offers several cooling advantages, including being small in size and lightweight, with a high heat transfer coefficient and the ability to bring about a decrease in the device’s heat gain [2].

The development of compact devices depends entirely on the success of the thermal management technology for progressive electronic devices; such types of devices must have a high performance, low weight, and small size. A solution is offered by the MCHS, which can resolve the problems of heat dissipation in current developing applications. The MCHS is a device that can be made to extract heat from the CPU. Therefore, MCHS has received significant attention from researchers in recent years.

Khonsue [3], for example, experimentally investigated the cooling performance of a MCHS made of aluminum. The width, length, and base thickness of the microchannel were 40, 28, and 2 mm. Deionized water was used as a base fluid. It was found that increasing the height of the channels and the speed of the water flow led to an increase in the heat transfer rate. Tran et al. [4] studied experimentally and numerically the pressure drop of the MCHS made from aluminum. The mass flow rates were found to range between 0.2 to 0.4 g/s, while the inlet water temperature was 25 °C. The results show that when the mass flow rate rises, the pressure drop increases. Ma et al. [5] experimentally investigated the heat transfer and the fluid flow of a 4-port heat sink with a zigzag and rectangular MCHS. The deionized water flow rate was found to range between 28 to 72 mL/min with an inlet temperature equal to 25 °C. It was found that at a low flow speed, the zigzag microchannel has a lower pressure drop than the rectangular microchannel, but when the flow increases, the pressure drop increases more in the zigzag microchannel than in the rectangular microchannel. Moreover, the heat transfer of the zigzag microchannel showed better results than the rectangular microchannel.

While the scales of MCHS devices are becoming smaller due to the rapid development of technology, the heat flux generated is seemingly increasing. In the nanotechnology field, recent developments have revealed a new set of heat transfer fluids, named nanofluids, such as those studied by Choi and Eastman [6] at the National Laboratory. Their research distinguished two principal advantages of using nanofluids: (1) they are comprised of milled particles or microparticles with a higher thermal conductivity than fluids; and (2) they have a better constancy.

Many researchers reported in their reviews that adding nanoparticles to the base fluid led to the increase in the thermal conductivity and viscosity of the nanofluids [7,8]. Nandhaumar and Senthilkumar [9] investigated the thermo physical properties of different nanoparticles such as ZnO, MgO, TiO_2_ and Al_2_O_3_ by using water and ethylene glycol as a base fluid. The volume fraction ranges between 0.5 to 2.5%. It was reported that, at a concentration of 2.5%, MgO-H_2_O has the highest thermal conductivity, with 7.4% enhancement over other nanofluids. Jeong et al. [10] investigated experimentally the thermal conductivity and viscosity of the ZnO nanofluid with a volume faction varying from 0.05 to 5%. Two different nanoparticle diameters of zinc oxide were used. The results show that the thermal conductivity improves up to 18% at the volume fraction of 5%. Lee et al. [11] studied the fluid thermal conductivity of CuO with nanoparticle diameters of 18.6 nm and 23.6 nm and Al_2_O_3_ with diameters of 24.4 nm and 38.4 nm suspended in two base fluids such as ethylene Glycol (EG) and water. They studied four different nanofluids; CuO in EG, CuO in water, Al_2_O_3_ in water, and Al_2_O_3_ in EG. They reported that the CuO/EG mixture showed 20% enhancement at 4 wt.% compared with other nanofluids.

Nanoparticle size is another factor which affects the viscosity and the thermal conductivity of the nanofluids. Alawi et al. [12] investigated experimentally and numerically the effect of nanoparticles size on the thermal properties of the nanofluids. They used different nanoparticles such as ZnO, SiO_2_, CuO and Al_2_O_3_ with diameter ranging from 20 nm to 100 nm. The results show that by decreasing the nanoparticles size led to the increase in the thermal conductivity of the nanofluids. Namburu et al. [13] investigated the effect of 20, 50, 100 nm diameter of SiO_2_ at 6% concentration using ethylene glycol and distilled water as a working fluid. They reported that the viscosity of the nanofluid increases when the nanoparticles size decreases. Eastman et al. [14] experimentally studied the effect of Cu nanoparticles concentration and diameter on the thermal conductivity of the nanofluids using ethylene glycol as a base fluid. It was noted the thermal conductivity enhanced with 40% at a concentration of 0.3% for nanoparticles diameters of 10 nm while at the concentration of 4% and for nanoparticles diameters of 23.6 nm the enhancement is 20%.

Other researchers reported that hybrid nanofluids could be considered as a next generation fluid. Esfe et al. [15] conducted an experiment for the measurement of the thermal conductivity of Cu/TiO_2_ suspended in 60% water and 40% EG. The nanofluid concentration varied from 0.1 to 2% and the nanoparticle diameter ranged between 40 and 70 nm. It was reported that, at temperature 30 °C, the thermal conductivity increased by 18.2% when the concentration increases to 2%. Hamid et al. [16] experimentally investigated five mixture ratios of TiO_2_:SiO_2_ (20:80, 40:60, 50:50, 60:40, and 80:20) nanoparticles which were suspended at a concentration of 1.0% in a solution of 60% pure water and 40% EG. The results indicated that all nanofluids show higher viscosity and thermal conductivity than the base fluid (EG/W). The mixture 20:80 (TiO_2_:SiO_2_) nanofluid shows the highest thermal conductivity compared to the other compositions, while for the ratio 50:50 (TiO_2_:SiO_2_) the nanofluid shows the highest viscosity. Siddiqui et al. [17] investigated the thermal properties of Cu/Al_2_O_3_ hybrid nanofluid with ratios 30:70, 50:50 and 70:30. The results show that the hybrid nanofluid with the ratio 50:50 has the best stability and the highest thermal conductivity among the other ratios. Many other researchers investigated the thermal properties of hybrid nanofluids by mixing different nanoparticles [18,19,20].

Mixing pure water with ethylene glycol was always commercially recommended as a heat transfer fluid for cooling or heating [21,22,23,24]. Namburu et al. [25] in 2007 have first reported results on the viscosity of a 40% of pure water and 60% of EG base fluid with CuO nanoparticles. Vajjha and Das [26] experimentally studied the effect of temperature on the thermal conductivity of CuO, Al_2_O_3_ and ZnO mixed with 60% ethylene glycol and 40% pure water. The test has been done for nanoparticle concentrations ranging from 0% to 10% and with temperature ranging from 298 K to 363 K. It was reported that the thermal conductivity of Al_2_O_3_ mixed with a base fluid of 60:40 EG/DW increased with 21% when the concentration increased to 6%. At a concentration of 10% the thermal conductivity of the nanofluid increased to 69% at temperature 365 K compared with pure water. Reddy and Rao [27] experimentally studied the thermal conductivity of TiO_2_ with a concentration varying from 0.2% to 1% and for nanoparticles a diameter of 21 nm. The nanoparticles were suspended in two different base fluids DW/EG (40%:60% and 50%:50%). Their results indicated that the thermal conductivity, for 1% concentration in base pure water, enhanced by 6% at temperature 30 °C. At the same concentration and temperature, the nanofluid thermal conductivity in base fluid EG/W (40%/60%) increased by 4.38%, while its increase is of 10.42% in a base fluid 50%/50% EG/W. Li and Zou [28] conducted an experiment to measure the thermophysical properties of SiC suspended in water/EG (60:40). HAAKE MARS III rheometer and KD2-Pro thermal analyzer were used to measure the viscosity and thermal conductivity of the nanofluid concentration varying from 0 to 1%. Their experimental results showed that, for a 1% nanofluid concentration, the thermal conductivity increased by 33.8% compared to the case of the base fluid, while the viscosity increased by 22.4%. Sundar et al. [29] studied the viscosity and the thermal conductivity of Al_2_O_3_ mixed with 20:80%, 40:60% and 60:40% of ethylene glycol and D water. The volume fraction is varying between 0.3 to 1.5%. It was reported that increase in the volume fraction concentration increased the thermal conductivity and the viscosity of nanofluid. The thermal conductivity enhanced with 32.26% for the fluid 20:80 EG/W in 1.5% volume concentrations and temperature 60 °C. The viscosity of 20:80 EG/W nanofluid appeared 1.29 times enhanced than for the other nanofluids at a volume concentration of 1.5% and temperature 0 °C. They also investigated the effect of Fe_3_O_4_ suspended in EG/DW (20:80%, 40:60% and 60:40%) [30]. They found that for Fe_3_O_4_ mixed with base fluid EG/DW (20:80), at temperature 20 °C, the thermal conductivity increased by 21.96% at the concentration by 2%.

The ultrafine suspended particles change the properties of transport and increase the heat transfer performance of the nanofluids as presented by Ahmed et al. [31]. However, the suspended particles with the usual slurries in millimeters or even micrometers may, at the end, cause several problems. For example, the abrasive action of the particles may clog or erode the pipelines. The fluid flow is accompanied by an increase in the pressure drop and additional rheological and instability problems can occur. As particles tend to settle rapidly, the slurries can provide better thermal conductivity. With the development of nanotechnology and nanoscience, the appearance of nanofluids has a great potential to enhance the means of thermal science, heat transfer and engineering challenges. Nonetheless, as the concept of nanofluids has been recently proposed, there are many unclear questions that need to be resolved [32,33].

In the past decade, due to their high thermal properties, the number of investigations related to nanofluids have increased. The interdisciplinary nature of research on nanofluids provides an insight into the opportunities to explore and discover nanotechnology frontiers [34]. The benefits of the use of nanofluids in computer technology are very promising.

Nguyen et al. [35] experimentally investigated the heat transfer performance of a computer cooling system. An Al_2_O_3_-water nanofluid was used as the cooling fluid inside the system. A 23% enhancement in the heat transfer coefficient of the nanofluid was found as compared with pure water. In addition, the temperature of the water block decreased, and the Nusselt number increased as the particle concentration increased. The heat transfer also increased, and the temperature decreased when the mass flow increased. Chein and Chuang [36] investigated MCHS performance using CuO-H_2_O with a volume fraction of 0.2 to 0.4%. They concluded that, compared with pure water, the nanofluid absorbed more thermal energy. Increasing the inflow rate showed no enhancement in the heat absorption. Since the nanoparticles of the nanofluid increased the viscosity of the fluid and the wall roughness, this behavior led to a slight increase in the pressure drop. Ho et al. [37] examined experimentally the convective force of the MCHS by using Al_2_O_3_-H_2_O as a working fluid with a volume fraction factor range from 0 to 2%. They reported that the nanofluid increased the density and thermal conductivity of the base fluid. The heat transfer coefficient showed a 70% enhancement in the MCHS performance when the nanofluid was used as a working fluid compared to pure water. They also discovered that nanofluids could reduce thermal resistance and the maximum wall temperature, which was a better result than pure water.

Tiwari et al. [38] experimentally investigated the heat transfer performance of the plate heat exchanger using different nanofluids such as CeO_2_/H_2_O, Al_2_O_3_/H_2_O, TiO_2_/H_2_O and SiO_2_/H_2_O with various concentrations. It was reported that CeO_2_/H_2_O nanofluid obtained the highest overall heat transfer coefficient ratio followed by Al_2_O_3_, TiO_2_, and finally SiO_2_. Haghighi et al. [39] experimentally studied the effect of nanofluid such as Al_2_O_3_, ZrO_2_ and TiO_2_ suspended in pure water on microtube performance. It was reported that the alumina and zirconia nanofluids show higher heat transfer coefficients at the same Reynolds number.

An experiment carried out by Rimbault et al. [40] studied the hydraulic and thermal fields of CuO-H_2_O inside a rectangular MCHS. They found that increasing the mass flow rate and the volume concentrations of nanoparticles led to an increase in the pressure drop of the nanofluid. The high temperature of the fluid led to a decrease in the viscosity of the fluid and afterwards a decrease in the pressure drop. They also found that, at low concentration, there was a slight heat transfer enhancement of 0.24% and 1.03%, respectively. However, at a concentration of 4.5%, a clear increase of heat transfer was found. Nazari et al. [41] examined the CPU performance by using different volume fractions of Al_2_O_3_ and CNT as a nanoparticle. 30% and 50% of ethylene glycol (EG) mixed with pure water was used as a based fluid. They found that mixing pure water with 30% of EG had a better cooling performance than pure water. The temperature reduced by about 22% when using CNT as a working fluid, but with the Al_2_O_3_ nanofluid, it dropped by 20%. The heat transfer enhanced by 6% and 13% when using 0.5% of Al_2_O_3_-H_2_O and 0.25% of CNT-H_2_O, respectively.

Sivakumar et al. [42] numerically and experimentally examined the performance of various nanofluids, such as Al_2_O_3_-H_2_O, CuO-H_2_O, and CuO-EG, in serpentine-shaped MCHS. The microchannels were made from copper with a hydraulic diameter ranging from 810 to 890 µm. The results showed that, due to the high density and viscosity, the CuO-EG had the highest transfer coefficient and pressure drop of all the nanofluids. Decreasing the diameter of the microchannels led to an improvement in the heat transfer coefficient and an increase in the pressure drop. Furthermore, Singh and Kumar [43] experimentally examined the fluid flow and heat transfer of wavy rectangular MCHS made by aluminum. To improve cooling performance, different concentrations of Al_2_O_3_ were mixed with water. They showed that increasing the Reynolds number and concentration of nanoparticles decreased the thermal resistance of MCHS and improved the overall heat transfer. A 3% volume concentration appeared at the highest pressure drop.

Arslan et al. [44] investigated the performance of a carbon nanotube (CNT) as a coolant in MCHS. They used a 0.01% weight concentration of carbon nanotube mixed with pure water. They found that, at a very low Reynolds number, the temperature difference of the carbon nanotube was higher than 30% as compared to pure water. The temperature difference between the CNT nanofluid and water were slightly similar when the flow rate increased. The pressure drop of CNT was close to the water in the case when the Reynolds number was less than 2000. A small addition of CNT to the base fluid led to an increase in the pressure to 1% at a low Reynolds number. However, increasing the Reynolds number led to a rise in the pressure of 8%. Increasing the Reynolds number was found to increase the pumping power. Thansekhar and Anbumeenakshi [45] studied the performance of aluminum MCHS using two types of nanofluids: Al_2_O_3_-H_2_O at a concentration of 0.1% and SiO_2_-H_2_O at a concentration of 0.25%. They stated that the heat transfer of Al_2_O_3_-H_2_O showed a 36.63% enhancement compared with pure water. Moreover, the nanofluid caused a slight pressure drop compared with pure water. Furthermore, the performances of different concentrations of TiO_2_-H_2_O in microchannels were experimentally investigated by Manay and Sahin [46]. The height of the microchannel was 200 µm and the Reynolds number varied from 100 to 750. The volume fraction ranged between 0.25% and 2.0%. It was observed that the heat transfer enhanced when the volume fraction reached 2.0 vol.%, then the heat dropped after 2.0 vol.%. In addition, they reported that the thermal resistance decreased when the size of the nanoparticles decreased. Increasing the concentration of the solids and the Reynolds number caused an increase in the Nusselt number.

Finally, it is clear to see from the previously outlined literature review that different nanofluids have been studied widely. However, research on the performance of computer cooling systems with Al_2_O_3_, ZrO_2_, and CeO_2_ suspended in 20% ethylene glycol and 80% distilled water have been very limited. Furthermore, there is no study that has yet observed the impact of these types of nanofluids on fluid flow and heat transfer in computer cooling systems. This paper will examine the cooling performance of a personal computer system with the addition of nanoparticles with different concentrations in base fluid at different atmospheric temperatures. A real computer setup with an LGA775 CPU socket has been used to find out the effect of the use of nanofluids and base fluid on the cooling system in real practical conditions. Table 1 presents the summary of experimental studies on the microchannel heat sink with nanofluids.

## 2. Nanofluid Preparation and Characterization

**Figure 1:**
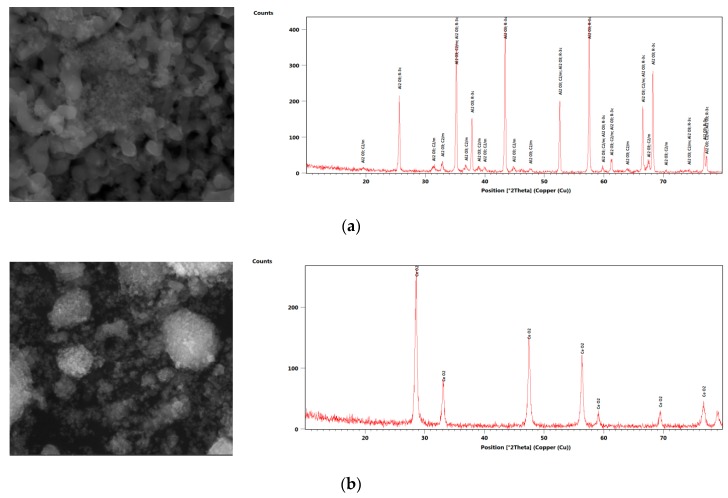

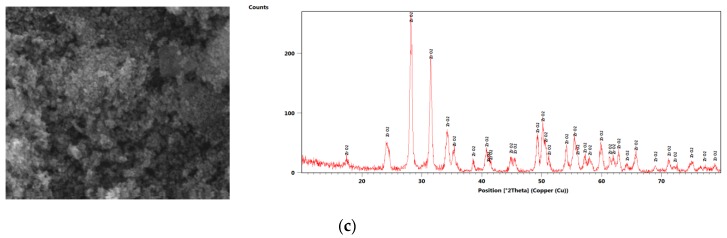
SEM and XRD of (**a**) Al_2_O_3_, (**b**) CeO_2_, (**c**) ZrO_2_ (Left figures show the SEM images and right figures show the XRD images with theta the reflection angle).

Aluminum oxide (Al_2_O_3_), zirconium dioxide (ZrO_2_), and cerium oxide (CeO_2_) nanoparticles were used for the preparation of a nanofluid, which was purchased from Skyspring Nanopowder and Nanoparticles (SSNANO). The properties of the nanoparticles can be seen in Table 2. The scanning electron microscopy (SEM) and X-Ray Diffraction (XRD) images of spherical-shaped CeO_2_, Al_2_O_3_, and ZrO_2_ nanoparticles are shown in Figure 1, taken directly after being received from the company. The SEM and XRD proved that all the nanoparticles are pure and have a diameter of less than 50 nm.

To prepare the nanofluids, a two-step method was used [24,28]. An electronic balance (AND-EJ610) was used to quantify the concentration of the nanoparticles with 0.5%, 1%, and 2%, respectively. The nanoparticles were suspended in 20% ethylene glycol and 80% distilled water using a heating magnetic stirrer. The beaker, which contained 600 mL of nanofluid, was placed on the magnetic stirrer and stirred for sixty minutes at 1100 rpm and a heat of 55 °C. The ethylene glycol was purchased from Sigma-Aldrich (Bucharest, Romania) and the distilled water was made in the laboratory. In order to decrease the agglomeration and maintain the stability of the nanoparticles in the base fluid, an ultrasonic probe mixer was used for one hour. This ultrasonic probe (Sonics and Materials, Newtown, CT USA) produce pulses with a maximum power of 500 Watts and 20 kHz frequency. Surfactants may reduce the thermal properties of the nanofluids; thus, no surfactants were added. In this work, the observation method was used, since it has been confirmed in previous works [47,48]. The CeO_2_, Al_2_O_3_, and ZrO_2_ nanofluids with concentrations of 0.5%, 1%, and 2%, respectively, showed stability for two weeks.

## 3. Thermophysical Properties

The transient hot-wire method is an operation mechanism applied to find out the thermal conductivity of nanofluids. In order to measure the thermal conductivity of the CeO_2_-EG/DW, Al_2_O_3_-EG/DW, and ZrO_2_-EG/DW nanofluids, a KD2 Pro thermal analyzer (Decagon Devices Inc., Pullman, WA, USA) was used with an accuracy of ∓5%. At a temperature of 25 °C, the thermal conductivity measurements were taken. At the same temperature, the viscosities of the nanofluids with a concentration varying from 0% to 2% were recorded using a Brookfield viscometer (DV-I prime) device. Moreover, the base fluid EG/DW (20:80) is considered to play a significant role in the density of the nanofluid. The size and the shape of the nanoparticles were ignored, since they have no effect on the density. A pycnometer was used to find out the density of the nanofluids. The measurements (thermal conductivity, density, and viscosity) were started with the base fluid EG/DW (20:80) for data verification and the results were compared with the DOW guide (Dow Chemical Company, Midland, MI, USA) [49] to validate the accuracy of the device and measurement procedure. All the measurements were recorded three times and the averages of these readings were used for analysis. However, the specific heat can be easily found based on Equation (1) [50].

(1) $$C_{nf}=\frac{\left(1-\varphi \right){\left(\rho C\right)}_{bf}+\varphi {\left(\rho C\right)}_{np}}{\rho_{nf}}$$

where $C_{nf}$, $C_{bf}$, $C_{np}$ represent the specific heat of the nanofluid, base fluid, and nanoparticles, respectively. $\rho_{nf},\;\rho_{bf},\;\rho_{np}$ represent the density of the nanofluid, base fluid, and nanoparticles, respectively. φ represent the nanoparticles concentration in the nanofluid.

Table 3 shows the thermophysical properties of Al_2_O_3_, CeO_2_, and ZrO_2_ nanoparticles suspended in EG/DW (20:80) with concentration 0.5%, 1%, and 2% at 25 °C.

## 4. Experimental Setup

**Figure 2:**
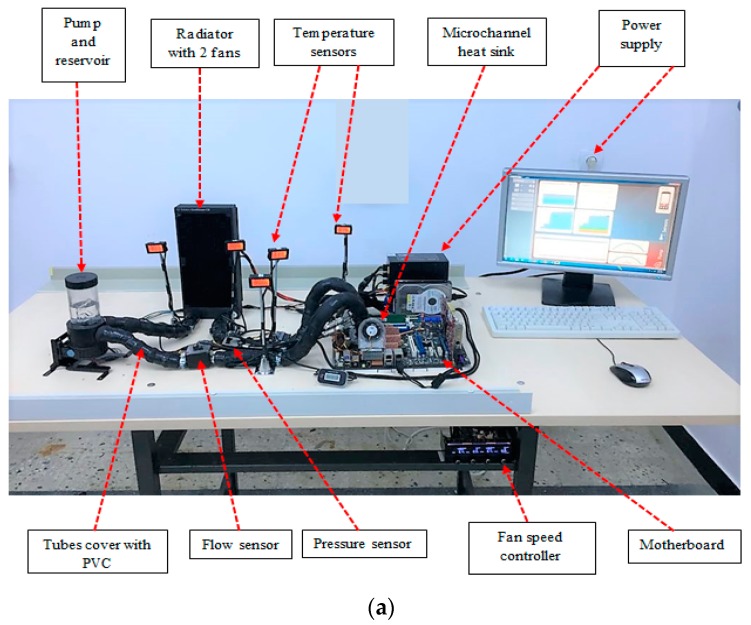

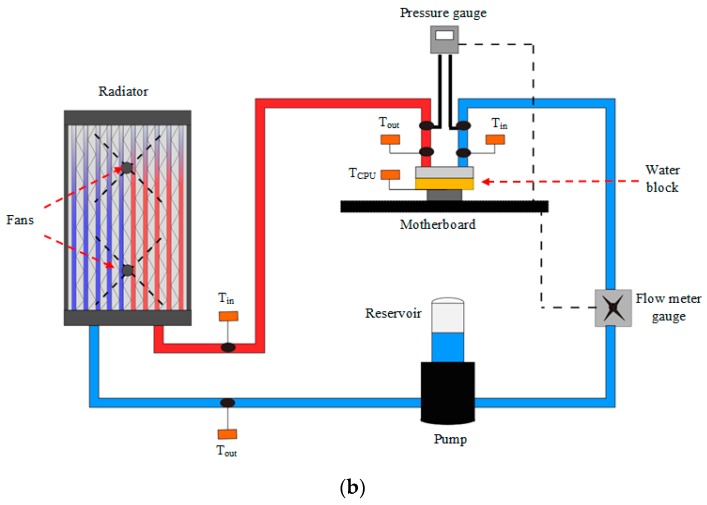
(**a**) The experimental setup, (**b**) The schematic diagram of the water-cooling system.

An experimental setup was created to investigate the thermal and heat transfer performances of the computer cooling system on the CPU using nanofluids and base fluid. The experimental setup with all components is shown in Figure 2a, while the schematic diagram of the complete experimental setup is shown in Figure 2b. The setup consists of a computer cooling system (water block, radiator, fans, pump, reservoir, tubes, fitting), motherboard (CPU, power supply, external hard), and sensors (pressure sensor, temperature sensors, flow meter sensor).

**Figure 3:**
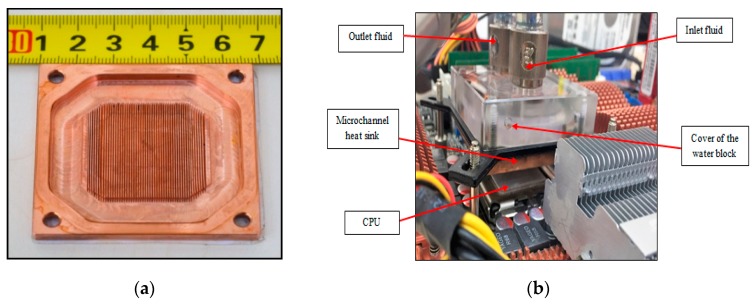

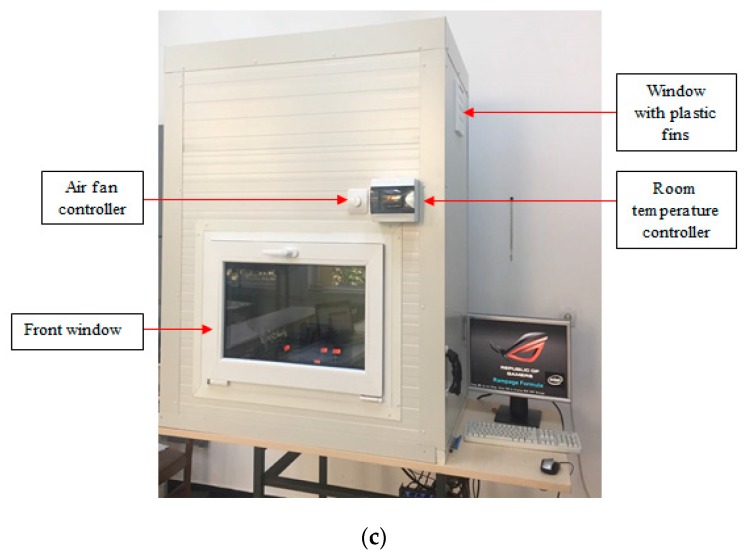
(**a**) Microchannel heat sink, (**b**) water block fixed above the CPU, (**c**) insulated room.

The computer cooling system type EK-KIT P360 (EKWB Company, Komenda, Slovenia) was used in this experiment. It consists of a water block, radiator, fans, pump, reservoir, tubes, and fitting. The dimensions of the water block are (57 × 57 × 22) mm^3^ and it is made of two parts. The cover is made from acrylic and the base is made from copper. The fluid inlet and outlet of the water block are fixed on the top of the cover. The copper base (microchannel heat sink) of the water block consists of 52 channels with a 0.3 mm width, 2 mm height, and 33 mm length, as seen in Figure 3a. 1500 L per hour is the maximum flow rate of the pump (D5 pump) with an integrated reservoir. It operates silently and produces less noise, since it has a special rubber shock absorber. Two fans with a maximum speed of 1200 rpm were fixed on the radiator. The radiator offers a large surface heat transfer, since the fins and tubes are made from copper with a 290 mL liquid capacity. To connect the main parts of the experiment, special tubes and fittings were used.

In the experiment, an ASUS X48 motherboard was used, since it offers the best hardware engineering, the most innovating ideas, and the fastest performance [51]. It also ensures higher overclock ability, so the frequency and voltage can be raised to produce more heat under operation. Through a land grid array (LGA) surface mount socket, the central processor unit (CPU) connects to the motherboard. The name of the socket is LGA775 as it contains 775 contacts arrayed with two cores [52]. The CPU top surface was covered with the thermal interface (8.5 W/(m·K)) to ensure equal distribution of the temperature on the surface, since it provides an effective heat transfer. In addition, a flat temperature sensor was fixed above the CPU interface to record the base heat sinks temperature of the water block. The water block was fixed above the CPU with four screws, as shown in Figure 3b. A fiberglass insulation room with a fan heater was built around the experiment to ensure that the ambient temperature was stable or could be increased. A glass window was placed on the front of the room to enable the temperature reading, as can be seen in Figure 3c.

The pump pushes the coolant to the water block with different mass flow rates, such as 0.028 kg/s, 0.056 kg/s, and 0.084 kg/s, respectively. The speed of the pump is controlled by a special controller made in the laboratory. The mass flow of the working fluid was recorded based on Aqua software. To examine the convection heat transfer and pressure drop, LCD temperature sensors and one pressure drop sensor are placed in the inlet and outlet of the water block. LCD temperature sensors have ability to measure the temperature between −40 °C and 70 °C. In this experiment, four temperature sensors with the T fitting (G1/4′) were fixed on the inlet of the water block and radiator. Another type of sensor is a flat sensor which is fixed between the CPU and water block. The sensitivity of the sensors is 0.1 °C. Both sensors have been calibrated prior to being used in this work. LCD temperature sensor are made by XSPS Company in Germany. All the experimental temperature readings have been recorded directly from the digital screen. The coolant leaves the water block towards the massive radiator in order to reject the heat and then the process was repeated. Rubber foam insulation was used to cover all the tubes to prevent any thermal contact with the atmosphere. The setup was run without any overclock from the CPU for twenty-four hours to ensure that the computer cooling system had a leak-proof circuit. After that, the heat flux was applied to the copper block by making stress with high overclocking over the CPU. The experimental study was repeated many times on different days to ensure the repeatability of the experiment. After the experiments, it was noted that that few nanoparticles sticking to the surface of the MCHS. However, the MCHS surface looked clean and without any corrosion after the measurements.

## 5. Data Processing

The most indicative parameter, at this work, is the heat transfer coefficient of the working fluid to the water block. It has been studied based on Newton’s cooling law [53];

(2) $$q=h\cdot A\cdot \Delta T_{m}$$

where *q* represents the heat generation rate in the processor, *h* represents the coefficient of the heat transfer, *A* represents the heat transfer area, and ∆*T_m_* represent the Log mean temperature difference between the MCHS walls and the working fluid which can be described below [53].

(3) $$\Delta T_{m}=\frac{T_{out}-T_{in}}{\ln\frac{T_{hsbt}-T_{in}}{T_{hsbt}-T_{out}}}$$

In which *T_out_* and *T_in_* are the fluid temperatures at the outlet and inlet of the water block, and *T_hsbt_* represents the heat sink base temperature that is considered constant.

Passing the working fluid through the water block leads to an increase in the temperature since the heat absorbed by it [53];

(4) $$q=\dot{m}\cdot C_{bf,nf}\left(T_{out}-T_{in}\right)$$

where $\dot{m}$ is the mass flow rate (kg/s), *C_bf,nf_* is the specific heat of base fluid and nanofluid (J/(kg·K)).

By equating Equations (2) and (4), the convective heat transfer coefficient can be written as [53]:

(5) $$h=\frac{\dot{m}\cdot C_{bf,nf}\left(T_{out}-T_{in}\right)}{A\frac{T_{out}-T_{in}}{\ln\frac{T_{hsbt}-T_{in}}{T_{hsbt}-T_{out}}}}$$

The pumping power has been calculated based on the below Equation [54];

(6) $$Pp=\frac{\dot{m}}{\rho_{bf,nf}}\Delta P$$

where $\dot{m}$ is the mass flow rate, $\rho_{bf,nf}$ is the density of the base fluid and nanofluid, ∆*P* is the absolute value of the pressure drop.

## 6. Results

### 6.1. Heat Sink Base Temperature

**Figure 4:**
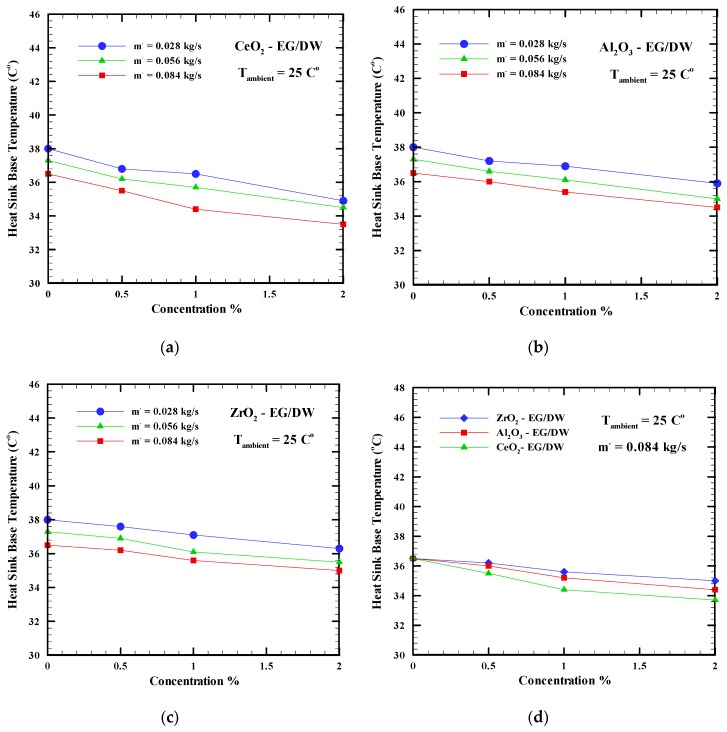
Heat sink base temperature versus nanoparticles concentrations at different mass flow rates for nanofluids (**a**) CeO_2_-EG/DW, (**b**) Al_2_O_3_-EG/DW, (**c**) ZrO_2_-EG/DW, (**d**) different nanofluids.

Minimizing the base temperature of the heat sink and the CPU temperature is the main objective of the computer cooling system. While a high heat flux was generated from the processor by increasing the load, the base temperature of the heat sink was measured. The heat sink base temperature was measured between the heated source and the water block which was used to evaluate the performance of the cooling system. A flat temperature sensor was placed between the base of the heat sink and the CPU. Figure 4a–c shows the heat sink base temperature versus the concentration of the Al_2_O_3_-EG/DW, CeO_2_-EG/DW, and ZrO_2_-EG/DW nanoparticles varying from 0% to 2%. During the test, three different mass flow rates of the nanofluids and base fluid were selected, such as 0.028 kg/s, 0.056 kg/s, and 0.084 kg/s. The ambient temperature was fixed at 25 °C. Moreover, the heat sink base temperatures related to the base fluid at different mass flow rates were shown as 0% concentration.

Figure 4a–c proved that adding any nanoparticles to the working fluid in the computer cooling system and increasing the mass flow rate led to larger heat removal from the heated block and kept the interface temperature minimized. This fact was noticed for all three nanoparticles used in this research. Figure 4a shows the effects of CeO_2_-EG/DW with different concentrations, such as 0%, 0.5%, 1%, and 2% on the heat sink base temperature. It can clearly be seen that increasing the mass flow rate to 0.084 kg/s and the concentration of the nanoparticles to 2% may drop the heat sink base temperature from 36.5 °C to 33.7 °C. Using Al_2_O_3_-EG/DW and ZrO_2_-EG/DW as a working fluid with the same mass flow rate and concentration can drop the temperature of the heat sink from 36.5 °C to 34.4 °C and from 36.5 °C to 35 °C, respectively, as shown in Figure 4b,c. At an ambient temperature of 25 °C, as shown in Figure 4d, the use of CeO_2_-EG/DW with a concentration of 2%, as a working fluid in the computer cooling system, decreased the base temperature of the heat sink more than in the case of the other nanofluids; the decrease in temperature is with 8.3% larger compared with the base fluid. Al_2_O_3_-EG/DW with 2% concentration and an ambient temperature of 25 °C showed a 6.1% decrease in temperature, while ZrO_2_-EG/DW showed a 4.2% decrease. Both results are compared with the base fluid at 0%. This happens because the high viscosity of the CeO_2_-EG/DW at a 2% concentration causes a hydraulic thickening and, in turn, the thermal boundary layer thickens. Consequently, CeO2-EG/DW shows the least heat sink temperature since it has the highest viscosity and thermal conductivity compared with other nanofluids.

**Figure 5:**
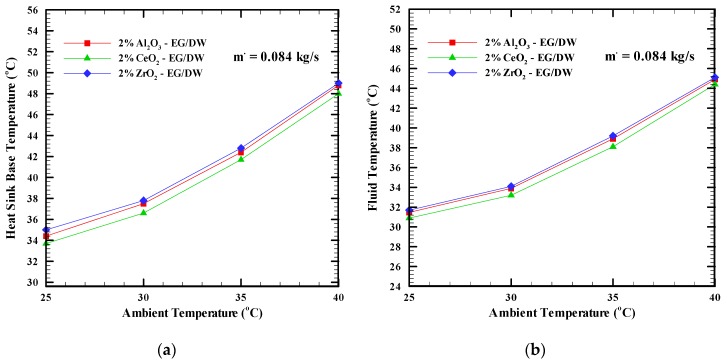
(**a**) Heat sink base temperature and (**b**) Fluid temperature, versus the ambient temperature for different nanofluids.

The effect of the ambient temperature varied from 25 °C to 40 °C on the heat sink base temperature and fluid temperature using nanofluids, such as CeO_2_-EG/DW, Al_2_O_3_-EG/DW, and ZrO_2_-EG/DW with 2% concentration and a mass flow rate of 0.085 kg/s shown in Figure 5. It can be clearly seen from the figures that increasing the ambient temperature causes an increase in the base temperature of the heat sink and fluid temperature for the base fluid and nanofluids. This happens because when the ambient temperature increases, the temperature of the processor increases which leads to an increase in the heat flux.

However, it should be noted that many researchers claim that increasing the mass flow rate by increasing the pump speed can cause unwanted noise. However, in this research, a D5 (PWM) pump was used which silently operates, even at a high speed, since it has a special rubber shock absorber.

### 6.2. Heat Transfer Coefficients

**Figure 6:**
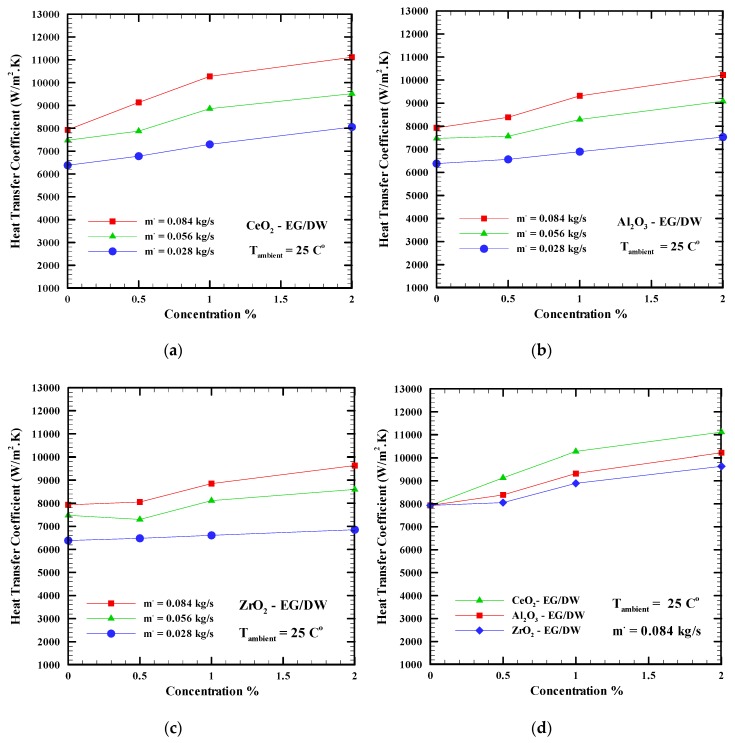
Heat transfer coefficient versus nanoparticles concentrations at different mass flow rates for nanofluids (**a**) CeO_2_-EG/DW, (**b**) Al_2_O_3_-EG/DW, (**c**) ZrO_2_-EG/DW, (**d**) different nanofluids.

The thermal performance of the nanofluids and how it affects the cooling system and the processor is the main objective of this research. Many researchers report that the thermal performances of nanofluids depend on the convective heat transfer coefficient. The heat transfer coefficient versus the concentration of the CeO_2_-EG/DW, Al_2_O_3_-EG/DW, ZrO_2_-EG/DW nanoparticles (0.5%, 1%, 2%) is shown in Figure 6a–d. The mass flow rates for the base fluid and nanofluids are 0.028 kg/s, 0.056 kg/s, and 0.084 kg/s, respectively, with an ambient temperature of 25 °C. A concentration of 0% is related to the base fluid EG/DW (20:80). Figures show that adding nanoparticles to the base fluid and increasing the mass flow rates lead to an increase in the heat transfer coefficient. This may happen because improving the Brownian motion and thermal conductivity of the working fluid may enhance the thermal performances of the nanofluid over the base fluid.

However, increasing the mass flow rate to 0.084 kg/s and the concentration to 2% shows the highest heat transfer coefficient. Figure 6a indicates that the heat transfer coefficient of CeO_2_-EG/DW increases from 7931 W/(m^2^·K) to 11,112 W/(m^2^·K) at a mass flow rate of 0.084 kg/s when the concentration of the nanoparticles increases from 0% to 2%. In addition, it increases from 7931 W/(m^2^·K) to 10,219 W/(m^2^·K) and from 7931 W/(m^2^·K) to 9634 W/(m^2^·K) when Al_2_O_3_-EG/DW and ZrO_2_-EG/DW are used as a working fluid, respectively (see Figure 6b,c). Figure 6d clearly indicates that CeO_2_-EG/DW at a concentration of 2% and an ambient temperature of 25 °C shows the highest heat transfer coefficient with a 29% enhancement compared with the base fluid, followed by Al_2_O_3_-EG/DW and ZrO_2_-EG/DW with enhancements of 22% and 17%, respectively. This behavior can be attributed to the high thermal conductivity and viscosity of the CeO_2_-EG/DW.

As the convection heat transfer coefficient is an increasing function of the thermal conductivity, Prandtl and Reynolds numbers (h (λ, Pr, Re)), one can observe that at any flow rate, with the increase in concentration, Pr and λ increase more than Re decreases due to the increase in viscosity, leading to an increase in the heat transfer coefficient. When the flow rate increases too, the enhancement of the heat transfer coefficient is more prominent.

**Figure 7:**
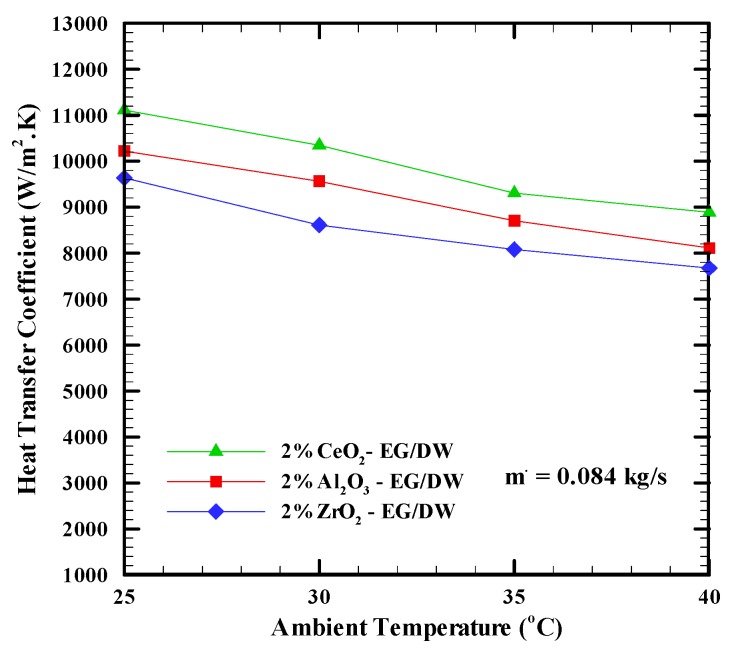
Heat transfer coefficient versus ambient temperature at for different nanofluids.

Figure 7 shows the effect of the variation of the ambient temperature on the heat transfer coefficient when Al_2_O_3_-EG/DW, CeO_2_-EG/DW nanofluids with a 2% concentration and a mass flow rate of 0.085 kg/s are used. It can be clearly seen for all nanofluids that increasing the ambient temperature leads to a decrease in the heat transfer coefficient. This is because the heat flux increases but the temperature difference between the cooling fluid and the heat sink base temperature increases more and for a specified constant heat transfer surface these corroborated effects lead to a decrease in the heat transfer coefficient (Equation (2)).

### 6.3. Pressure Drop

**Figure 8:**
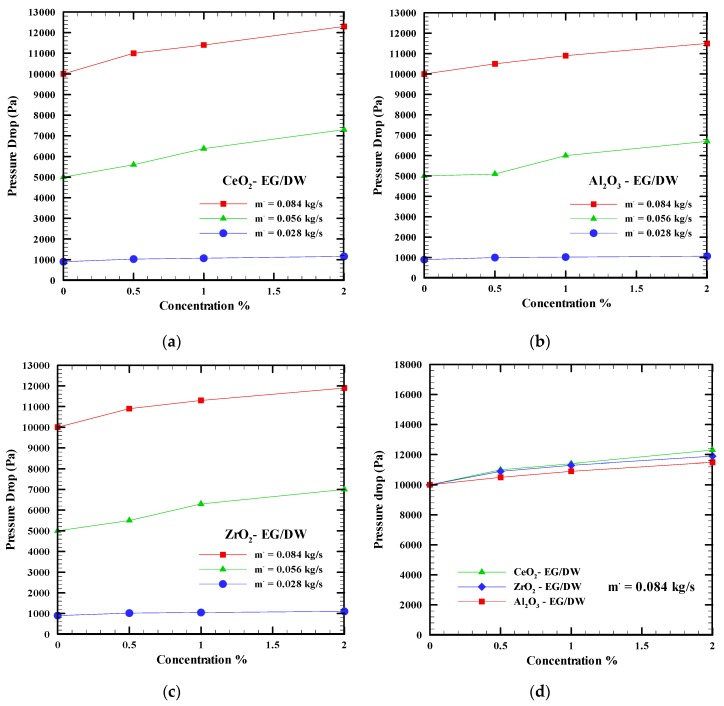
Pressure drop versus nanoparticles concentrations at different mass flow rates for nanofluids (**a**) CeO_2_-EG/DW, (**b**) Al_2_O_3_-EG/DW, (**c**) ZrO_2_-EG/DW, (**d**) different nanofluids.

To ensure the benefit of nanofluids in the cooling process, the pressure drop must be investigated, since nanoparticles have been added to the base fluid. The pressure drop occurs when the working fluid passes through the water block and then to the narrow microchannels. Based on the Aqua software, Figure 8a–d show the pressure drop of the base fluid and nanofluids (CeO_2_-EG/DW, Al_2_O_3_-EG/DW, ZrO_2_-EG/DW) for concentrations varying from 0% to 2% and mass flow rates varying from 0.028 kg/s to 0.084 kg/s. Generally, from all the figures, it can be demonstrated that the pressure drops in the water block increases when the mass flow and nanoparticle concentration of the nanofluids increase. This phenomenon happens because adding nanoparticles to the base fluid increase the density and cinematic viscosity of the fluid which leads to an increase in the pressure drop; with the increase in the mass flow rate, and consequently of the fluid velocity (w), the pressure drop grows more (ΔP(w,μ)).

However, Figure 8d shows that when the concentration increases from 0% to 2% for all nanofluids, the pressure drop shows a slight increase. Al_2_O_3_-EG/DW shows the lowest pressure drop followed by ZrO_2_-EG/DW and CeO_2_-EG/DW. This is due since Al_2_O_3_-EG/DW has a lower viscosity than the other nanofluids. The CeO_2_ and ZrO_2_ nanofluids show very close results at a concentration of 0.5%, but the difference increases when the concentration increases to 2%.

### 6.4. Pumping Power

**Figure 9:**
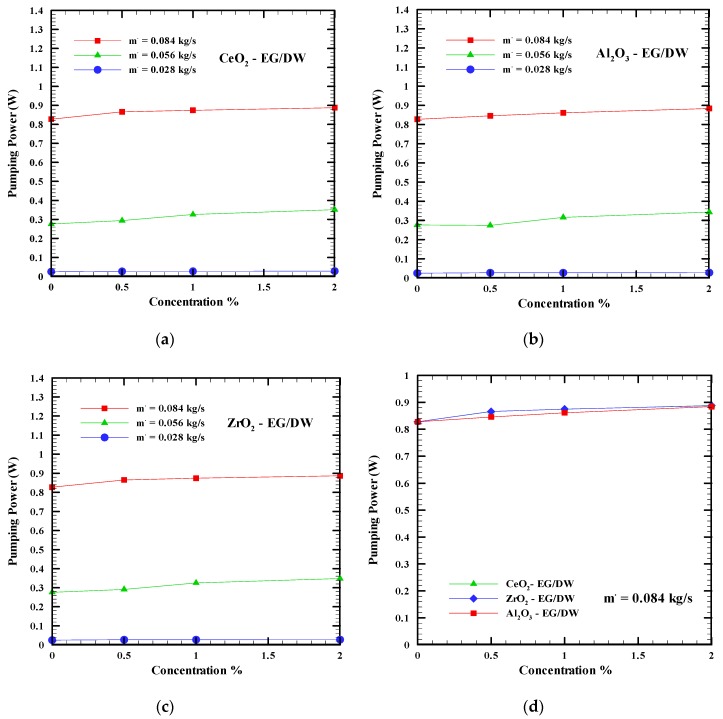
Pumping power versus nanoparticles concentrations at different mass flow rates for nanofluids (**a**) CeO_2_-EG/DW, (**b**) Al_2_O_3_-EG/DW, (**c**) ZrO_2_-EG/DW, (**d**) different nanofluids.

Using nanofluids in computer applications as an effective heat transfer working fluid should enhance the heat transfer characteristics without much increase in pumping power. When the working fluid passes over the micro heat sink channels, a pressure drop occurs. The system requires more pumping power to overcome this pressure drop. Figure 9a–d show the pumping power versus nanoparticle concentrations of the nanofluid at different mass flows, such as 0.028 kg/s, 0.056 kg/s, and 0.084 kg/s. It was shown that adding nanoparticles to the base fluid causes a slight increase in the pumping power, that is balanced by the better thermal performance of the nanofluid compared to the base fluid. Nonetheless, Al_2_O_3_-EG/DW shows the lowest pumping power, while CeO_2_-EG/DW and ZrO_2_-EG/DW show very close results to each other. The pumping power follows the trend of the pressure drop, which is amplified by the measure of the fluid velocity (Equation (6)).

## 7. Conclusions

In this research, the effects of using different types of nanofluids in computer cooling systems have been experimentally investigated. Three CeO_2_, Al_2_O_3_, and ZrO_2_ nanoparticles were used suspended with 20% ethylene glycol and 80% distilled water. The setup and the procedure of the experiment were described in detail. Parameters, such as CPU temperature, ambient temperature, heat transfer coefficient of the base fluid and nanofluids in MCHS, the pressure drop of base fluids and nanofluids in MCHS, and pumping power of the MCHS, have been experimentally investigated. The results point out the following:

- Increasing the concentration of the nanoparticles from 0.5% to 2% and increasing the mass flow rate causes a decrease in the base temperature of the heat sink more than the base fluid. The CeO_2_ nanofluid at a 2% concentration reduces the temperature with an 8.3% since the CeO_2_ nanofluid has the highest thermal conductivity and viscosity. At the same concentration, the Al_2_O_3_ and ZrO_2_ nanofluids show 6.1% and 4.2% temperature decrease, respectively, compared with the base fluid EG/DW (20:80). Moreover, increasing the ambient temperature from 25 °C to 40 °C led to an increase of the heat sink base temperature.
- Adding nanoparticles to the base fluid and increasing the mass flow rate leads to an increase in the heat transfer coefficient much more than the base fluid. The CeO_2_ nanofluid shows the highest heat transfer coefficient with a 29% enhancement, while the Al_2_O_3_ nanofluid shows a 22% enhancement. The ZrO_2_ showed a 17% enhancement. Furthermore, increasing the ambient temperature from 25 °C to 40 °C led to a decrease in the heat transfer coefficient.
- The pressure drops and pumping power increase when the mass flow rate and concentration of the nanofluid increases. The Al_2_O_3_-EG/DW shows the lowest value followed by ZrO_2_-EG/DW and CeO_2_-EG/DW. However, a slight increase of pumping power and pressure drop can be balanced by considering the high improvement of the nanofluid in computer cooling performance compared to the base fluid.
- The novelty of the study consists in providing information about the comparative behavior of CeO_2_, Al_2_O_3_ and ZrO_2_ nanoparticles suspended in EG/DW (20:80) when used as cooling fluids in computer cooling systems. Graphs are provided with the variation of the convective heat transfer coefficient, heat sink base temperature, pressure drop and pumping power, against the nanoparticles concentration in nanofluids and the mass flow rate of the cooling fluid.
- From an academic point of view, the work establishes a procedure for the preparation and characterization of nanofluids. An experimental setup was created to investigate the heat transfer performances of a computer cooling system with nanofluids as the cooling fluid. Of academic interest is the data processing developed in the work.

## Figures and Tables

**Table 1:** Summary of experimental studies on microchannel heat sink (MCHS) with nanofluids.

Author	N.P Type	N.P Size (nm)	Base Fluid	V.F %	MCHS Enhancement
Nguyen et al. [35]	Al_2_O_3_	47	Water	0.69 to 4.5	23% enhancement in heat transfer coefficient of nanofluid
Chein and Chuang [36]	CuO	20 to 80	Water	0.2 to 0.4	Nanofluid absorbed more thermal energy than pure water. Increasing in flow rate shows no enhancement in the heat absorption.
Ho et al. [37]	Al_2_O_3_	33	Water	0 to 2	70% enhancement in heat transfer coefficient.
Korpyś et al. [38]	CuO	30 to 50	Water	0.0086 to 0.0225	Nanofluid slightly improved the heat transfer performance
Nitiapiruk et al. [39]	TiO_2_	---	Water	0.5, 1 2	Nusselt number rise between 13% and 20% when the volume concentration increases between 0 and 2%.
Rimbault et al. [40]	CuO	29	Water	0.24, 1.03, 4.5	At low concentration, slight heat transfer enhancement with 0.24% and 1.03%, although at concentration 4.5% a clear increase of heat transfer was found
Nazari et al. [41]	Al_2_O_3_ CNT	40	Water EG	0.1, 0.25, 0.5	Mixing the pure water with 30% of **ethylene Glycol** (EG) has better cooling performance than pure water Temperature decreased about 20% by using Alumina nanofluid and 22% in the case of **carbon nanotube** (CNT).
Sivakumar et al. [42]	Al_2_O_3_ CuO	15	Water EG	0.01 to 0.3	Decreasing the microchannels diameter is led to improve the heat transfer coefficient and increasing pressure drop.
Singh and Kumar [43]	Al_2_O_3_	---	Water	1 to 3	Increasing in concentration of nanoparticles and Reynolds number lead to decrease the thermal resistance of MCHS.
Arslan et al. [44]	CNT	---	Water	0.01	At very low Reynolds number, the temperature difference rang of carbon nanotube is higher with 30% as compared to pure water.
Thansekhar and Anbumeenakshi [45]	Al_2_O_3_ SiO_2_	43	Water	0.1, 0.25	The heat transfer of Al_2_O_3_-H_2_O shows 36.63% enhancement compere with pure water.
Manay and Sahin [46]	TiO_2_	25	Water	0.25, 0.5, 1, 1.5, 2	Heat transfer enhanced when the volume fraction reached to 2.0 vol.% then the heat drop after. The thermal resistance decreases when the nanoparticles size decreases.

**Table 2:** The properties of Al_2_O_3,_ ZrO_2_, and CeO_2_ nanoparticles.

Nanoparticle	Purity	Diameter	Density	Shape	Used Nanoparticles Concentrations (%)
CeO_2_	99.9%	<50 nm	7.22 g/cm^3^	spherical	0.5, 1, 2
Al_2_O_3_	99.9%	<50 nm	3.97 g/cm^3^	Nearly spherical	0.5, 1, 2
ZrO_2_	99.9%	<50 nm	5.6 g/cm^3^	Nearly spherical	0.5, 1, 2

**Table 3:** Thermo-physical properties of the nanofluids at temperature 25 °C.

Working Fluid	Concentration %	K (W/m·K)	C_p_ (J/(kg·K))	$\rho \;(\mathrm{kg}/m^{3})$	µ (Ns/m^2^)
Base fluid	0	0.498	3828	1029	0.00147
CeO_2_ EG/DW	0.5	0.527	3713	1057	0.00159
	1	0.535	3605	1089	0.00173
	2	0.547	3406	1152	0.00188
Al_2_O_3_ EG/DW	0.5	0.525	3771	1040	0.00153
	1	0.532	3717	1058	0.00166
	2	0.545	3612	1086	0.00178
ZrO_2_ EG/DW	0.5	0.503	3737	1047	0.00158
	1	0.509	3650	1076	0.00171
	2	0.518	3487	1119	0.00187
